# Missing data estimation in fMRI dynamic causal modeling

**DOI:** 10.3389/fnins.2014.00191

**Published:** 2014-07-04

**Authors:** Shaza B. Zaghlool, Christopher L. Wyatt

**Affiliations:** Bradley Department of Electrical and Computer Engineering, Virginia TechBlacksburg, VA, USA

**Keywords:** dynamic causal modeling, expectation-maximization, missing data

## Abstract

Dynamic Causal Modeling (DCM) can be used to quantify cognitive function in individuals as effective connectivity. However, ambiguity among subjects in the number and location of discernible active regions prevents all candidate models from being compared in all subjects, precluding the use of DCM as an individual cognitive phenotyping tool. This paper proposes a solution to this problem by treating missing regions in the first-level analysis as missing data, and performing estimation of the time course associated with any missing region using one of four candidate methods: zero-filling, average-filling, noise-filling using a fixed stochastic process, or one estimated using expectation-maximization. The effect of this estimation scheme was analyzed by treating it as a preprocessing step to DCM and observing the resulting effects on model evidence. Simulation studies show that estimation using expectation-maximization yields the highest classification accuracy using a simple loss function and highest model evidence, relative to other methods. This result held for various dataset sizes and varying numbers of model choice. In real data, application to Go/No-Go and Simon tasks allowed computation of signals from the missing nodes and the consequent computation of model evidence in all subjects compared to 62 and 48 percent respectively if no preprocessing was performed. These results demonstrate the face validity of the preprocessing scheme and open the possibility of using single-subject DCM as an individual cognitive phenotyping tool.

## Introduction

The quantitative measurement of cognitive function (cognitive phenotyping) is a central task in computational psychiatry (Montague et al., 2012) and related proposals (Miller, 2010). The functional systems perspective (Bullmore and Sporns, 2009) is emerging as a promising approach to identifying such phenotypes, leveraging the theory and analysis methods of network science and dynamical systems. A leading method in this dynamical systems approach is Dynamic Causal Modeling (DCM) (Friston et al., 2003). DCM models the interaction among brain regions as a dynamical system, giving rise to a generative model of brain activity that is augmented with an observation model that depends on the imaging modality. Inversion of the model provides an estimate of the model parameters and supports the comparison and averaging of models. DCM has had extensive development with several variations, improvements, and applications. The reader is referred to recent reviews (Daunizeau et al., 2011) for more details.

DCM makes a strict separation between where activity occurs and how that activity is coordinated. When fMRI is used as the imaging modality, DCM uses a first-level statistical analysis to locate active regions for the task or contrast under consideration. This first-level analysis determines which voxels have a time-course that correlates with some known pattern of stimulation by using a general linear model to separate stimulus induced signals from noise. When performed subject-wise, this commonly reveals variation in the activation among individuals, occurring as missing, or extra regions of activation relative to the proposed model topology (Bennett and Miller, 2010). The sources of this variation are complex including individual anatomical, functional, and measurement factors that are not related to the cognitive task. However, factors such as cognitive strategy which are important for cognitive phenotyping have also been identified (Van Horn et al., 2008).

While the number of nodes resulting from the first-level analysis is itself informative in focal neurological disease (e.g., Cabeza, 2002), other diseases are thought to be related to connectivity or other parameters of the generative DCM model. For instance, in schizophrenia, DCM analysis showed significantly decreased bilateral endogenous connectivity between two regions in comparison to healthy controls (Wagner et al., 2013). What is unclear in any given disease is whether the number of active nodes itself is a sufficient diagnostic indicator, or whether DCM model parameters add significant information. For example, suppose a DCM for a specific condition has been identified. To apply the model to an individual, their fMRI data is first subjected to a first-level test to identify active regions. Now, suppose that the subject does not have an active region close to that expected. Is this due to a false-negative in the first-level analysis or is this model simply not applicable in any way to this individual?

This is a common issue faced when using DCM. For example, differences in topology and dynamics have also consistently been shown between younger and older adults (Madden et al., 1999; Reuter-Lorenz et al., 2000; Grady, 2002), with evidence that older adults recruit bilateral frontal brain areas when performing difficult cognitive tasks, while younger subjects tend toward unilateral processing (Cabeza et al., 1997, 2003, 2004). For the specific task studied in this paper, the Go/No-Go task, age-related effects on network structure have been found using independent component analysis (Stevens et al., 2007, 2009). These and numerous other studies show that variations in topology at the subject level relevant to cognitive phenotyping exist.

Existing methodologies (Penny et al., 2003a,b, 2010; Stephan et al., 2009; Rosaa et al., 2010) generally discard subjects with missing regions and any extra regions of activation in an individual subject in order to proceed with DCM. For population-based studies, where inferences are generally at the group level, this approach poses few serious concerns, as it is assumed these sources of variability result in identically distributed additive noise. However, to use DCM at the subject-level, for example to classify the subject from model parameters (Brodersen et al., 2013) or by model selection (Stephan et al., 2009), this presents a problem, since there is no way to specify the node's location in this subset of subjects, and hence to extract the node's signal (principle eigenvector) from the fMRI data.

The first-level analysis is susceptible to false negatives (the underlying problem we are addressing here) due to multiple comparisons and the associated corrections. Control of false positives and negatives is a critical step in the interpretation of statistical parameter maps of activation. Typically, to reduce the chance of false positives, a conservative (corrected) *p*-value can be used. Alternatively, to reduce false negatives a loose or relaxed *p*-value can be used. Although not explicitly stated, this technique appears to be common practice when applying DCM when nodes are missing and dropping subjects is undesirable, in particular for exploratory analysis. When an expected node does not appear, the corrected *p*-value can be relaxed in stages until activation is produced at or close by the expected location. Although *ad-hoc* in nature, we can place this idea on a more sound theoretical base by treating the missing node as missing data, a common problem in statistics and machine learning, giving rise to numerous approaches (Pedro et al., 2010).

Using this observation, this paper presents a preprocessing approach to allow DCM to be conducted in single subjects by treating missing regions of activation, relative to a candidate model, as missing data (section Estimation of Missing Data). Four methods for estimating the missing data are presented, modeling the missing signal in progressive sophistication. These methods are compared using simulated networks in section Simulated Dataset Emulating a Go/No-Go Task. Application to representative phenotyping tasks is described in section Real Datasets and analyzed in section Real Dataset(s) Results, showing the missing data approaches enable classification of all subjects using DCM, letting the model evidence rather than the number of first-level nodes alone be the determining factor for phenotyping. We conclude with a comparison to using a relaxed statistical threshold in the first-level analysis.

## Materials and methods

### Dynamic causal modeling (DCM)

DCM is a method for estimating parameters of a generative model of neural activity, and comparison of those models. DCM for fMRI involves a bilinear model for neurodynamics and an extended Balloon model for the hemodynamics. This bilinear approximation reduces the parameter to three sets: the intrinsic connectivity, the modulatory connectivity, and connectivity of direct inputs. The main focus of DCM analysis is usually on the changes in connectivity embedded in the bilinear parameters. For a full description of the DCM variant used here, see Friston et al. (2003).

DCM differs from other approaches such as structural equation modeling and autoregression models where one assumes the measured responses are driven by intrinsic noise processes (McIntosh and Gonzalez-Lima, 1994). DCM not only accommodates the bilinear and dynamic aspects of neuronal interactions, but accommodates experimentally designed inputs making the analysis of effective connectivity more similar to the analysis of region-specific effects. In contrast to task-related fMRI DCM, recent work has also presented DCM as a tool for the investigation of directional brain connectivity in resting state fMRI (Friston et al., 2013b).

In this paper, DCM is used in two different ways. First, to compare the methods with a ground-truth, the forward model of DCM is used to simulate plausible synthetic data (described in section Simulated Dataset Emulating a Go/No-Go Task). Following that DCM inversion (which also uses the forward model) is used to perform the model estimation (described in section fMRI Model Specification and Statistical Analysis) and is applied to both the synthetic and real fMRI data.

### Estimation of missing data

Simple methods to the missing data problem involve substituting a constant for the missing feature, either zero (henceforth Zero-filling) or the mean value from samples with the data available (Mean-filling). Another approach is to fill the missing data with a noise process whose parameters mimic the missing data. Here we use an independent identically distributed Gaussian process using either constant mean and variance (Noise-filling) or those estimated given the available data for the individual using expectation maximization (EM-estimated).

The EM algorithm maximizes the log-likelihood of the available data, with the missing data marginalized so that the log-likelihood for the full data (available plus missing) is greater than that for available data alone. Here, the available data is the principle eigenvector of the nodes that match the model and survived the first-level activation test, while the missing data is that from nodes in the model with no corresponding activation. See Supplementary Material for a full description of the EM algorithm.

These various methods are increasingly sophisticated mathematical representations of missing data. Zero-filling and mean-filling are both examples of constant-filling. This is the simplest approach we could envision to allow model inversion and comparison in the case of missing nodes. However, they have a similar effect on the dynamics and, given infinite data, should converge to the same model parameters during estimation. Neither is a particularly good approximation as one would not expect a constant signal arising from neural activity, however, they are included to provide a baseline for comparison of the noise-filling methods, i.e., we would not expect these methods to perform better than more sophisticated models. We note that using the mean-filling approach is equivalent to using a more informative prior in the inversion process and would be expected to perform better given limited data.

The noise and EM-filling approaches are similar in that both fill with independent identically distributed noise, the former with a fixed variance, the latter with a variance estimated from the remaining data using EM. These approaches seek to mimic the unstructured noise (measurement and physiological) while not adding additional information. This is similar to using an artificially low *p*-value in the first-level analysis to find a corresponding node location, but without the confound of signal content perhaps unrelated to the task.

### Simulated dataset emulating a Go/No-Go task

A network was designed to replicate a standard Go/No-Go task based on extensive literature (e.g., Stevens et al., 2007). Networks were used to simulate realistic BOLD time series using the methods and code described in Smith et al. (2011) and FMRI Analysis Group (2011). The simulations were based on the DCM fMRI hemodynamic forward model (Friston et al., 2003), which uses the nonlinear balloon model for the vascular dynamics, on top of a causal model.

A network simulating a Go/No-Go task was generated. The task consisted of a visual and auditory stimulus. Absence of the auditory stimulus is accompanied with a motor function while the presence of the auditory stimulus inhibits the motor function. The visual input *u*_1_ and the auditory input *u*_2_ together formed the DCM input *u*. Input *u*_1_ consisted of a box function of zeros and ones resembling two different visual inputs. Input *u*_2_ also consisted of a box function representing the absence/presence of an auditory stimulus. The output of 0 represented the “No-Go” response and 1 represented the “Go” response. Different plausible (i.e., acceptably valid) networks were designed (Supplementary Figure 1).

The simulated Go/No-Go task involved four brain nodes: a visual node (V), an auditory node (A), a motor node (M), and a prefrontal cortex node (P). Input *u*_1_ was connected to V, *u*_2_ was connected to A, and output was measured at M.

The inputs (visual and auditory) were Poisson processes with the rate controlled by the presence or absence of the stimulus. The auditory node had an external binary input and was generated based on a Poisson process that controls the likelihood of switching state. Neural noise/variability of standard deviation 1/20 of the difference in height between the two states is added to the signal. The mean durations of the states were 2.5 s (up) and 10 s (down), with the asymmetry representing longer average “no beep” than “beep” durations. This external input into the auditory node is viewed as a signal feeding directly into the auditory node.

The visual node also had an external binary input and that was generated based on a Poisson process that controls the likelihood of switching state. Neural noise/variability of standard deviation 1/20 of the difference in height between the two states was added to the signal. The mean durations of the states were 5 s (up) and 5 s (down), representing the input durations. This external input into the visual node is viewed as a signal feeding directly into the visual node.

In the bilinear DCM state equation ż = (α*A* + ∑*u_j_B_j_*)*z* + *Cu*, the non-diagonal terms in *A* determine the network connections between nodes. To model the within-node temporal decay, the diagonal terms in *A* are all set to −1. Consequently, the term α is the rate of change of activity of mass-neuronal processing within a region (which inhibits itself) as described in Smith et al. (2011). The effect of the within-node dynamics (exponential temporal decay) is to create a lag between the input and output of every node. Although the original DCM forward model (Friston et al., 2000) includes a prior on α that results in a mean 1 s lag between neural time series from directly connected nodes, this unrealistically long lag was originally coded into DCM for practical algorithmic purposes in the Bayesian modeling. Even though this is not a problem when DCM is applied to real data, it produces unrealistic lags in a simulation based on this model. Therefore, it is changed to a more realistic time constant of a mean neural lag of approximately 50 ms as in Smith et al. (2011).

Following that, every node's neural time series was fed through the nonlinear balloon model for vascular dynamics and this allowed it to respond to changing neural demand. The amplitude of the neural time series was set so that the amount of nonlinearity (with respect to both changing neural amplitude and duration) matched what is seen in typical 3T fMRI data, and BOLD % signal change amplitudes of approximately 4% resulted (relative to mean intensity of simulated time courses). The balloon model parameters were set according to the prior means in DCM. There are differences in hemodynamic processes across brain areas and subjects which lead to different lags between the neural processes and the BOLD signal. Variations that occur could be up to 1 s or more (Handwerker et al., 2004; Chang et al., 2008). This was taken into account by adding randomness to the balloon model parameters at each node, producing variations in the HRF (hemodynamic response function) delay of standard deviation 0.5 s. Lastly, thermal white noise with a standard deviation 0.1–1% (of mean signal level) was added.

The BOLD data was sampled with a TR of 3 s (simulation output sampling rate), and the simulations included 100 realizations/subjects all using the same simulation parameters, having randomized external input time series, randomized HRF parameters at each node and slightly randomized connection strengths. Each subject's data was a 10-min fMRI session (200 time points) and the time-step of the integrator was 5 ms.

The networks consisted of four nodes with two external inputs, and connection strengths were randomized to have mean 0.4 and standard deviation 0.1 (with maximum range limited to 0.2:0.6). Each four node network can also be represented as a 4 × 4 connection matrix where each element (i, j) determines the presence of a connection from node i to node j.

In the simulation, iterative methods were used for the approximation of solutions of the differential equations. The fourth order Runge-Kutta method was used to integrate the differential equations. After simulating DCMs all through to the hemodynamic model to get ROI time series for the subjects, the next step was feeding the simulated time series into statistical parametric maps (SPM) for estimation. The main aim in doing the simulations was to use the simulated time series to create the disconnected time series and then use EM-estimation to fill in the missing nodes. At that point, different models can be tested to see the evidence relative to the true simulated model.

Using the simulated ROIs of 10 subjects, 16 DCMs were specified in SPM having the configurations shown in Supplementary Figure 1 (Total 160 subject-model pairs). Following that, the DCMs were estimated using the Variational Bayes algorithm in SPM. The default SPM options were generally used in all runs (SPM 8).

For each subject with a given model, one of the four nodes was randomly removed keeping track of the original model label. Then the missing node was replaced with alternative versions of EM-estimated, Noise-filling, Mean-filling, and Zero-filling time series. The noise that was used for noise-substitution consisted of minimal white noise with standard deviation 0.05% (of mean signal level).

In the case where there was a missing node, output from the missing node was first replaced by zeros. For instance, if the second node was missing, then the output *y*(t) = [*y*_1_(*t*), *y*_2_(*t*), *y*_3_(*t*), *y*_4_(*t*)] was replaced by *y*(*t*) = [*y*_1_(*t*), 0, *y*_3_(*t*), *y*_4_(*t*)]. In another run, the output from the missing node was replaced by the average value of that region from other subjects where that node is available. In a third run, the output from the missing node was replaced by the white noise. In a fourth run, the output from the missing node was replaced by the EM algorithm estimated value taken from the true case. The complexity of EM is much greater in comparison with the other methods (magnitude of 1000× runtime). However, the added overhead it takes to run the DCM is approximately 0.001% and this computation is purely a preprocessing step.

Following that, DCM model comparison was performed in SPM and the relative evidence was computed for the 16 models for each subject. Classification of a particular subject was then based on locating the highest model evidence. The results of the classification based on SPM's model evidence computations were compared with the known truths about the underlying models. The results are shown and discussed in section Simulation Results. The same sort of analysis is also shown when randomly removing two out of the four nodes.

### Real datasets

#### Data acquisition

Two previously collected and publicly available raw datasets were used for the real dataset experiments with relevant details repeated here. The first fMRI dataset (Xue et al., 2011) was collected using a 3T Siemens Allegra MRI scanner. For each of two runs, 182 functional T2^*^-weighted echo-planar images (EPI) were acquired with the parameters described in Xue et al. (2011). Additionally, a T2-weighted matched bandwidth high-resolution anatomical scan (same slice prescription as EPI) and MPRAGE were acquired. Stimulus presentation and timing of all stimuli and response events was done using Matlab.

The first dataset consisted of 21 healthy native English-speaking subjects (age ranged from 18 to 39). All subjects had normal or corrected-to-normal vision and were right handed. A manual stop-signal paradigm was used where a task consisted of a number of Go trials and Stop trials. On Go trials, the subject responded as fast as possible to the visual stimulus presented on a screen. For this manual task, subjects responded to the letters T or D with their right index or middle finger respectively. On Stop trials (which represented 25% of trials), the subject tried to stop his/her response when hearing a stop signal (a beep) which was played at a particular stop-signal delay (SSD) after presenting the visual stimulus.

The second fMRI dataset (Kelly and Milham, 2011) was collected using a 3T Siemens Allegra MRI scanner. For each of two runs, 151 functional T2^*^-weighted EPI were acquired. Additionally, a T1-weighted high-resolution anatomical scan was acquired. Details are in Kelly and Milham (2011).

The second dataset consisted of 21 healthy native English-speaking subjects performing a rapid event-related Simon task. In each trial, a red or green box appeared on the right or left side of the screen. Participants used their left index finger to respond to the presentation of a green box and their right index finger to respond to the presentation of a red box. In congruent trials the green box appeared on the left or the red box on the right, while in more demanding incongruent trials the green box appeared on the right and the red on the left. Subjects performed two blocks, each containing 48 congruent and 48 incongruent trials, presented in a pre-determined order, interspersed with 24 null trials (fixation only).

#### fMRI preprocessing

All preprocessing was done using SPM8 (Friston et al., 2013a). The functional images were first realigned to remove any movement artifacts from the fMRI time series, followed by slice-timing correction and co-registration of the first scan from each session to the first scan of the first session. Then the images within each session were aligned to the first image of the session. Co-registration was done between the structural and mean functional data, maximizing the mutual information (MI). Following that, SPM was used to segment the structural image using the default tissue probability maps as priors. Gray matter, white matter, and bias-field corrected structural images were created. After registration, these maps represented the prior probability of different tissue classes being found at each location in an image. The final processes included spatial normalization of the functional data followed by smoothing of the data by an 8 mm kernel.

#### fMRI model specification and statistical analysis

Categorical responses were modeled using the stimulus onset times and movement parameters from the realignment stage. Three conditions were specified for the first dataset, namely, “Go,” “StopFail,” and “StopSucc.” The GLM design matrix specified for this dataset included two sessions and has one row for each scan and one column for each effect or explanatory variable (i.e., regressor or stimulus function). As for the second dataset, four conditions were specified—“Congruent Correct,” “Congruent Incorrect,” “Incongruent Correct,” and “Incongruent Incorrect.” Each session had those four conditions with the appropriate stimulus onset times for each. The GLM design matrix specified for the second dataset also included two sessions.

Estimation of the GLM parameters was done using a Bayesian approach (using a VB algorithm) (Friston et al., 2002). The Bayesian approach allows one to specify spatial priors for regression coefficients and regularized voxel-wise AR(P)models for fMRI noise processes. This algorithm does not require the functional images to be spatially smoothed. A model order of 0 was selected which corresponded to the assumption that the errors were IID. This assumption could affect the results as there might be functional relationships among voxels in the same region.

After estimation, contrast vectors were applied to the results to produce SPMs or posterior probability maps (PPMs) and tables of statistics. Since null events were not explicitly modeled, they constituted an implicit baseline. The following t-contrasts were defined for the first dataset—Go-Null, StopSucc-Null, StopFail-Null, and StopSucc-Go. As for the second dataset, the following t-contrasts were defined: Congruent-Incongruent and Correct-Incorrect. Regions were identified where there was a high probability (level of confidence) that the response exceeded a particular size (i.e., signal change). Note this is quite different from classical inference, in which low probabilities of the null hypothesis that the size of the response is zero, are sought.

## Results and discussion

### Simulation results

**Figure 1:**
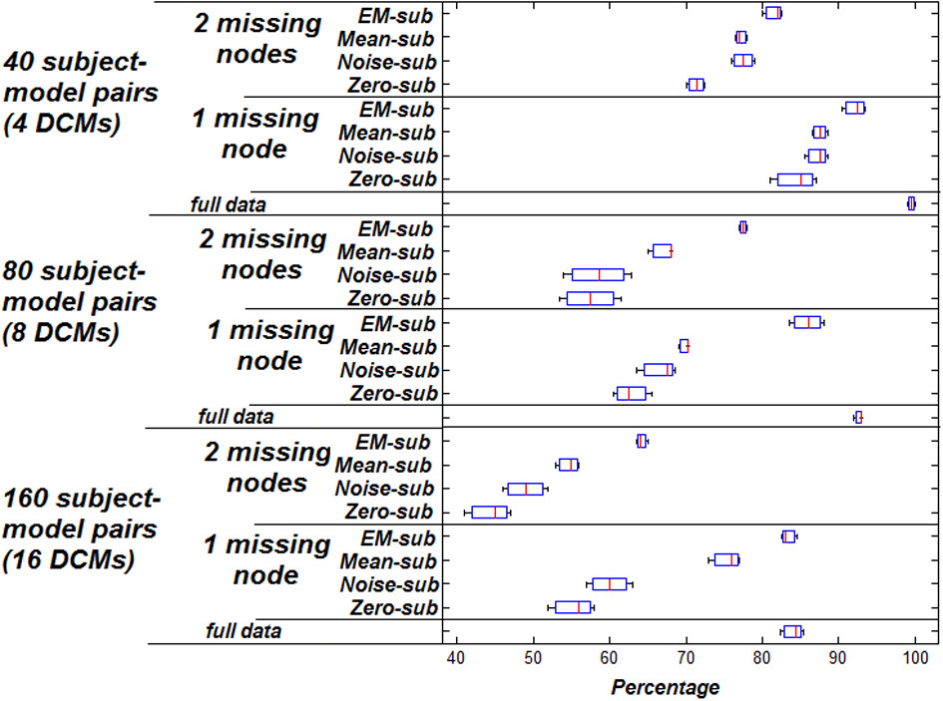
**Accuracy of classification based on highest model evidence vs. ground truth.** Zero missing nodes (full data) is the baseline for comparison. Box plots show the bootstrap variance of classification accuracy.

Figure 1 shows the classification accuracy based on highest model evidence vs. ground truth using the 160 simulated subject-model pairs described in section Estimation of Missing Data. This figure shows the percentage at which the true underlying model could be recovered for the different techniques. Taking a subset of the 16 DCMs (8 instead of 16 including the true model), the same kind of analysis was performed and the results are shown in the same figure. Again, taking an even smaller subset of the original 16 DCMs (4 instead of 16 including the true model), the same analysis was performed and those results are shown.

As shown, using the EM-algorithm for filling in missing data yields the highest classification accuracy relative to the other methods. This holds for various dataset sizes and varying numbers of model choice. Having two missing nodes instead of one causes a reduction in the performance, indicating that a greater proportion of missing data negatively affects the estimation process. This is intuitive since there is a severe decrease from mean value of 83.125–63.75% when dropping a second node in the case of EM substitution, and a similar trend for other methods. Also, when comparing having the full simulation data to having one or two missing nodes, it is noted that classification accuracy increases, given more available data.

**Figure 2:**
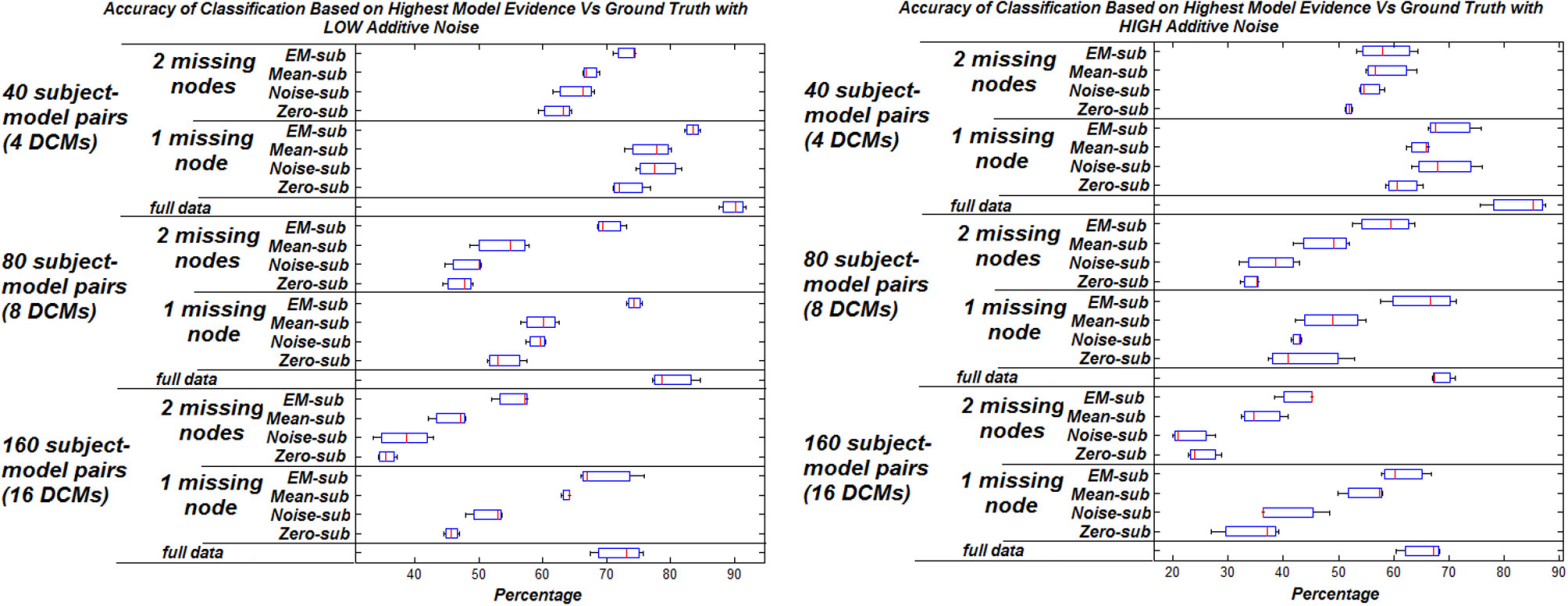
**Accuracy of classification based on highest model evidence vs. ground truth with varying levels of noise.** Zero missing nodes (full data) is the baseline for comparison. Box plots show the bootstrap variance of classification accuracy.

The effect of increasing the noise added to the signals on the model selection was also studied. Two levels of additive noise, relative to the signal to noise ratio, were examined—low noise (standard deviation of 0.001) and high noise (standard deviation of 0.01) results in Figure 2. There is a general decrease in the percentage of correctly identified models corresponding to systematically increasing the additive noise.

In general, the highest classification rate was obtained when using the EM algorithm compared to the other three filling methods. The computational complexity of EM, however is much greater, adding to the overall analysis run time (approximately 1000 times longer). This is considered trivial however as it is only fractions of a second and resulted in a better classification rate.

Most available fMRI datasets range in subject counts from tens to hundreds. Simulated fMRI data is much easier to obtain in abundance and the time consumed in data collection can be drastically reduced. Also, varying experimental factors can be done more efficiently and the data can be resynthesized, recollected, and reanalyzed. However, the problem of using simulated data is that since one cannot confidently determine the cause behind node variation, the simulated models might not necessarily follow the same natural paths of real data, and may contain certain experimental biases as a result of imposing certain properties in the simulation. Thus, the simulated dataset was only used a preliminary test of the proposed solution to missing nodes in DCM. The simulations have shown a significant improvement in subject classification when using the EM algorithm as a method to estimate missing nodes. The ability to perform subject classification presents an advantage to the solution presented in this paper in the light of previous work where such types of analyses were avoided.

Model selection based on the highest evidence is subject to several factors such as the number of models being compared, how similar each of the models is to the other models being considered in the same comparison, and the signal-noise ratio of the data. When performing model selection, reducing the number of models being compared can improve the accuracy of model selection. Also, including models that are farther (based on lower evidence) can improve the accuracy of model selection. In this context, model selection was based on Bayesian Model Comparison which is a relative measure. Since the complete model space can be rather large, model comparison including the entire model space would be unpractical.

In these simulations, model selection was based on the relative comparison of evidence values for each model. However, by comparing the evidences of different models and performing model selection/classification by choosing the highest evidence, an assumption of a 0–1 loss is being made. A value of 0 is given if the predicted output is the same as the actual output, and a value of 1 if the predicted output is different from the actual output. This means that although a certain model might have higher evidence than another one, it might not necessarily be the most rational one. There may be occasions when one model clearly dominates the others and other occasions when the choice is misleading. Choosing the correct model would require the consideration of costs which can be included using a certain utility function. A 0–1 loss function loss function may not be a good choice in a medical scenario because it should be expected for the cost of an incorrect decision to be different from a correct one, i.e., an incorrect decision should have a higher penalty than a correct one.

The classification decision in the simulations was evaluated under different utilities that could be possible in a clinical case. Two different models were generated (representing diseased/healthy states) with prior probabilities for each model taken from a training population. Given a new subject, the model evidence was estimated for each model, using the priors and the utilities. The prior probabilities were made realistic using asymmetric utilities (the cost of correct vs. incorrect diagnosis was different). The sensitivity of the improvement using EM filling was thus tested. This allows the impact/significance of improvement to be more emphasized when using utilities and priors that are relevant to the real world.

To the best of our knowledge, missing data approaches have not been used to overcome this problem in DCM. The results presented in this section indicate the validity of the proposed solution. This is also taken a step further by testing these methods on real fMRI data.

### Real dataset(s) results

Variational Bayes was used for the estimation of parameters for each DCM in the real subjects. To measure the difference between the computed parameters before and after estimation of missing data, for each time series where the full model could be estimated, the Root Mean Square Error (RMSE) was used. RMSE is a quantitative measure of the difference between the SPM computed parameters with the full data, and the computed SPM parameters vector after estimating the missing data. The initial estimated parameter vector is defined as: θ = {*A, B, C, h*} where the BOLD parameters are *h* = {τ, α, *E*_0_, *V*_0_, τ_*s*,_ τ_*f*_, ϵ}. $\hat{\theta }$ is the estimated parameters vector after computing the missing data. The RMSE is defined as:

$$RMSE=\sqrt{\frac{1}{N}\sum {\left(\hat{\theta }-\theta \right)}^{2}}$$

Similarly, the MI was computed between the initial BOLD signal and the estimated BOLD signal after missing data estimation. The MI is a method of measuring the interdependence of two random variables. If two signals are truly independent, then the MI will be zero. The MI of two discrete random variables *x* and *y* can be defined as:

$$MI\left(x,y\right)=\sum_{y}\sum_{x}p\left(x,y\right)log\left(\frac{p(x,y)}{p\left(x\right)p(y)}\right)$$

Volume of interest (VOI) time series were based on centers of peak activation. VOIs were sphere shaped with a radius of 8 mm. The peak activation locations were used to place spheres to extract the principle eigenvectors as the nodes' signals. Four nodes were typically involved in this particular task for each subject (Stevens et al., 2007). The *p*-value was manually adjusted to drop nodes (using a more conservative *p*-value). Any extra nodes were ignored for all subjects. Missing nodes were estimated using zero-substitution, mean-substitution, and EM-estimation. The parameters were estimated for all subjects. Estimation of parameters was done using VB. The MI was computed between the predicted and measured response. The RMSE was measured between the initial and estimated parameters.

MI increases when using better estimation methods i.e., mean-substitution vs. zero-substitution and EM-substitution vs. mean-substitution. RMSE error decreases when using better estimation methods i.e., mean-substitution vs. zero-substitution and EM-substitution vs. mean-substitution. The more missing nodes there are that need to be estimated, the greater the error RMSE and the lower the MI.

**Figure 3:**
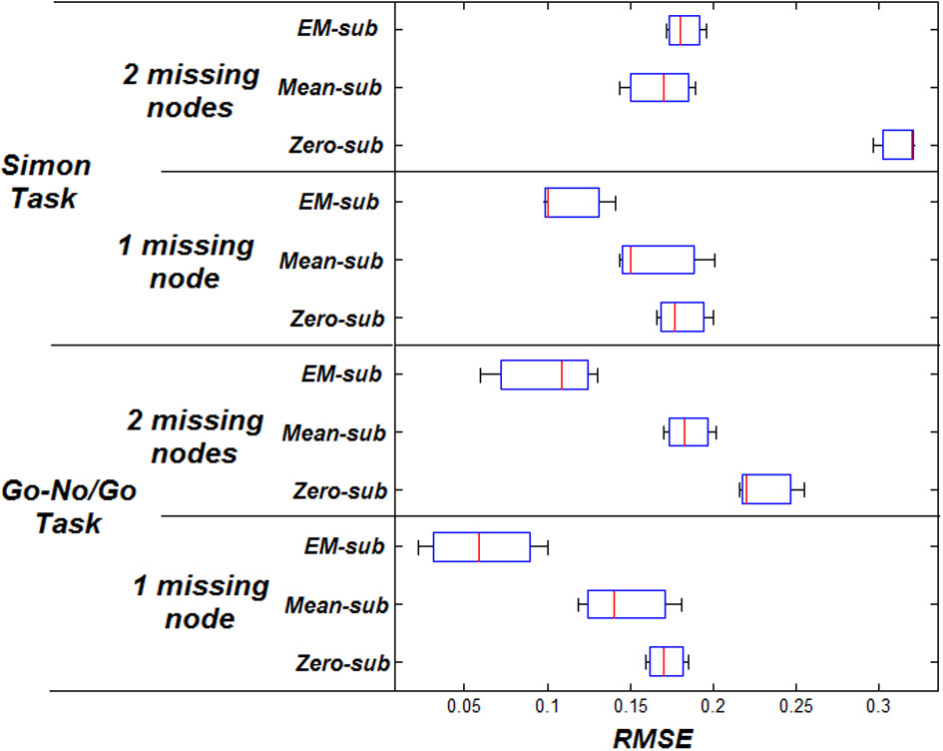
**RMSE between initial and final parameters for both datasets**.

**Figure 4:**
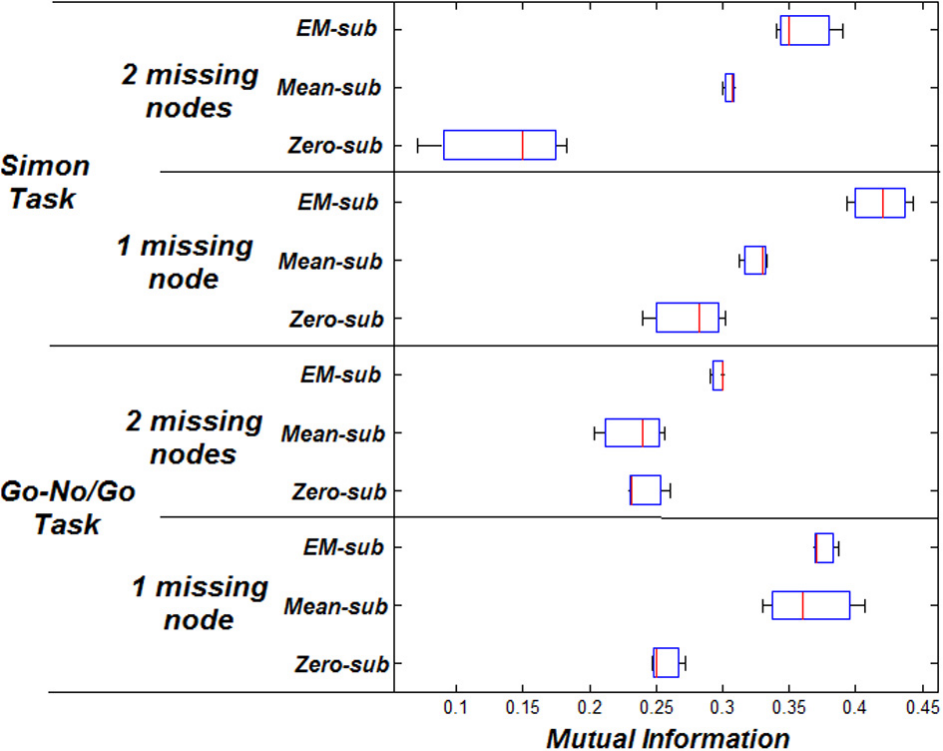
**MI between predicted and measured response for both datasets**.

The same comparison between estimated and actual time-series and parameters was also performed for the Simon task dataset. The VOI time series were also based on centers of peak activation and were sphere shaped with a radius of 8 mm. However, in this dataset, three nodes were considered ideal for each subject. The effect on the RMSE and MI for both datasets can be shown in Figures 3, 4 respectively.

### Cluster analysis

The ability to obtain similar results to the simulation experiments in the real experiments is desirable. However, since classification experiments by nature need the ground truth to be predetermined, and such information is not available, unsupervised methods such as clustering are the only option. Clustering allows the formation of certain groupings and the properties/parameters of the clusters can be used to study the effect of missing node estimation.

In order to perform the same sort of classification tests in real data, some kind of ground truth must be available. Because the classification of real subjects from any dataset would essentially have no ground truth, the parameter space was explored by looking at the variation/groupings in the estimated parameters for the population. Groupings were compared by looking at the distance between cluster centers by using different values of *k* in *k*-means clustering. This gave an idea about the potential variation that would exist in a population if a classifier were to be used. The aim of clustering the parameters was to validate the pattern of results obtained from the simulation experiments. It was expected that there would be an increase in the between cluster scatter and a decrease in the within scatter of clusters if there was in improvement.

For all the subjects from the Go/No-Go dataset, the parameters were estimated using VB. Clustering was then performed on the estimated parameters from all the subjects. That was followed with some statistical analysis on the clustered parameters including computations of the mean, mode, and standard deviation.

**Figure 5:**
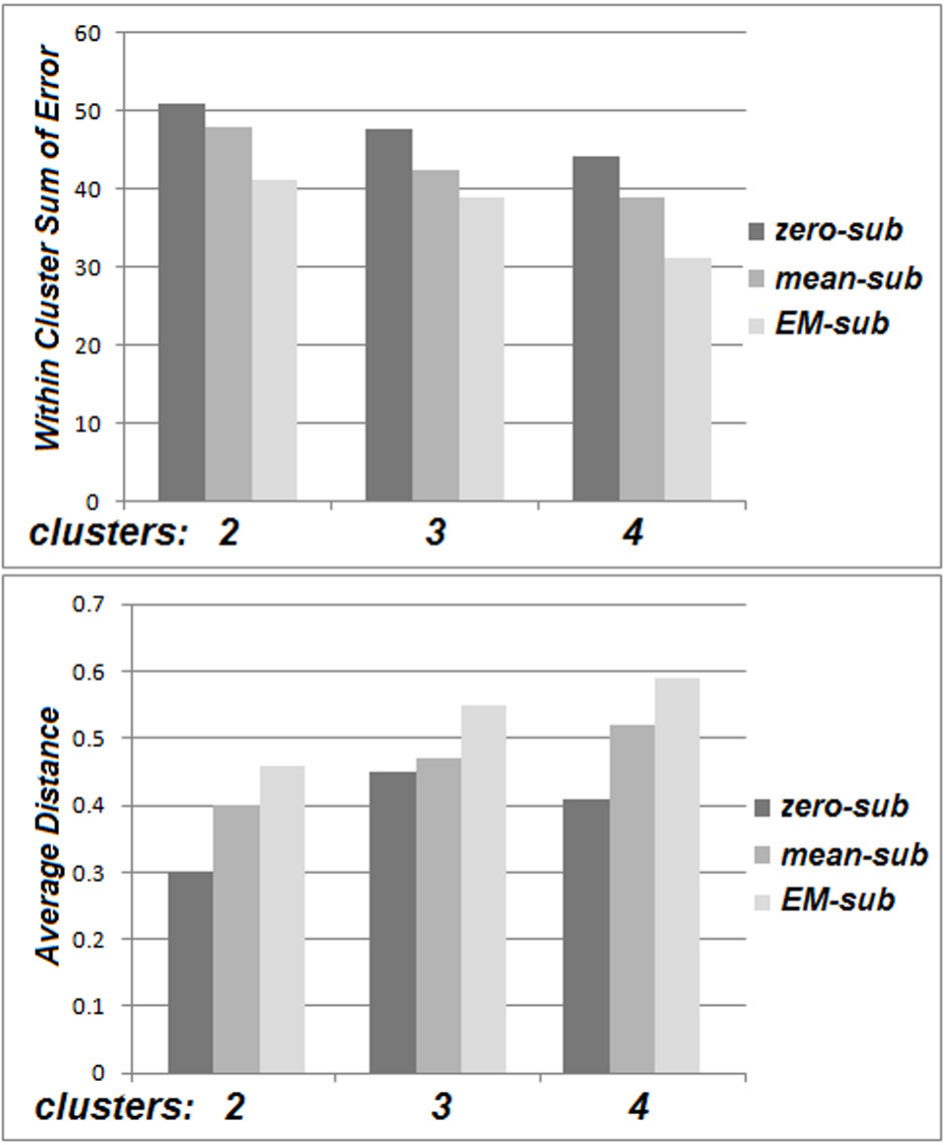
**The within cluster sum of squared errors (top) and average distance between cluster centers for the Go No/Go dataset (bottom)**.

The within cluster sum of squared errors was higher for zero-substitution in comparison with mean-substitution, and higher for mean-substitution with respect to EM-estimation. Also, a comparison of distance between cluster centers for different values of *k* (averaged over all parameters and all clusters) was performed. The distance between cluster centers is based on several factors. An increase in the number of clusters, spreads them out, decreasing the distances between them. The distance between cluster centers was smaller for zero-substitution in comparison with mean-substitution and smaller for mean-substitution with respect to EM-estimation. These patterns can be deduced from Figure 5.

**Figure 6:**
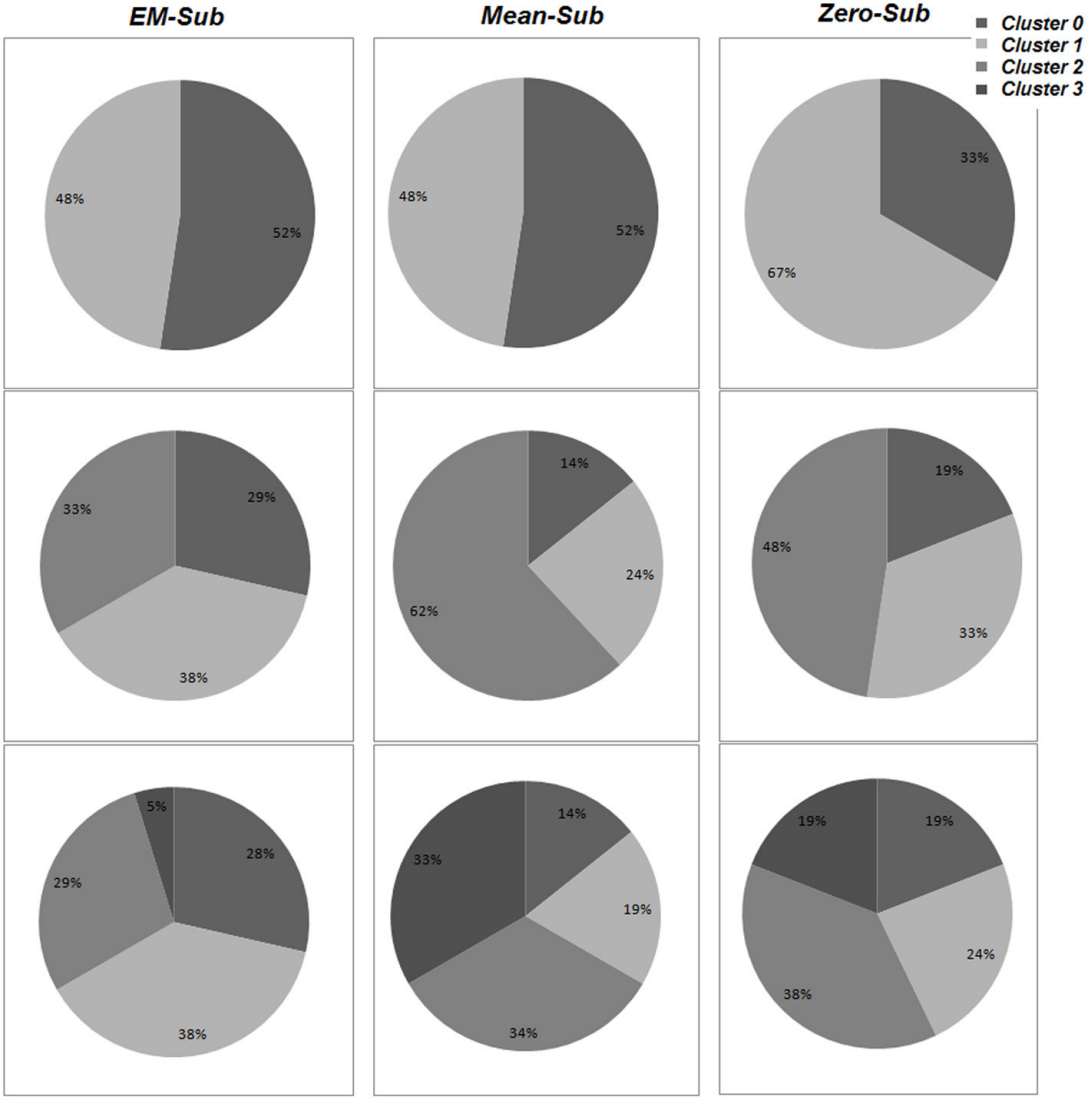
**The percentage of population belonging to each cluster for varying values of k, using all three methods (EM-sub, Mean-sub, and Zero-sub).** This is shown using VB for parameter estimation.

Details of the cluster composition are shown in Figure 6. The specific cluster compositions might not indicate more than that changing the number of clusters obviously changes each cluster composition and the determination of population distribution as a whole. However, the formed cluster groupings for the various *k*-values are indications for variations existing among these populations. Since these variations can be grouped, this would allow the usage of classification to identify or label an unknown subject based on the formed groups/clusters.

### Finding nodes using a less conservative p-value

It is important to determine how increasing the *p*-value to locate missing nodes compares to using EM to estimate those missing nodes in real data. We explore whether the evidence is higher (in model comparison) for a missing node that was EM estimated or for that same node to be located by systematically increasing the *p*-value until a noise point eventually emerges nearby (nearest Euclidean distance). This helps in determining at what point this technique becomes noise.

**Figure 7:**
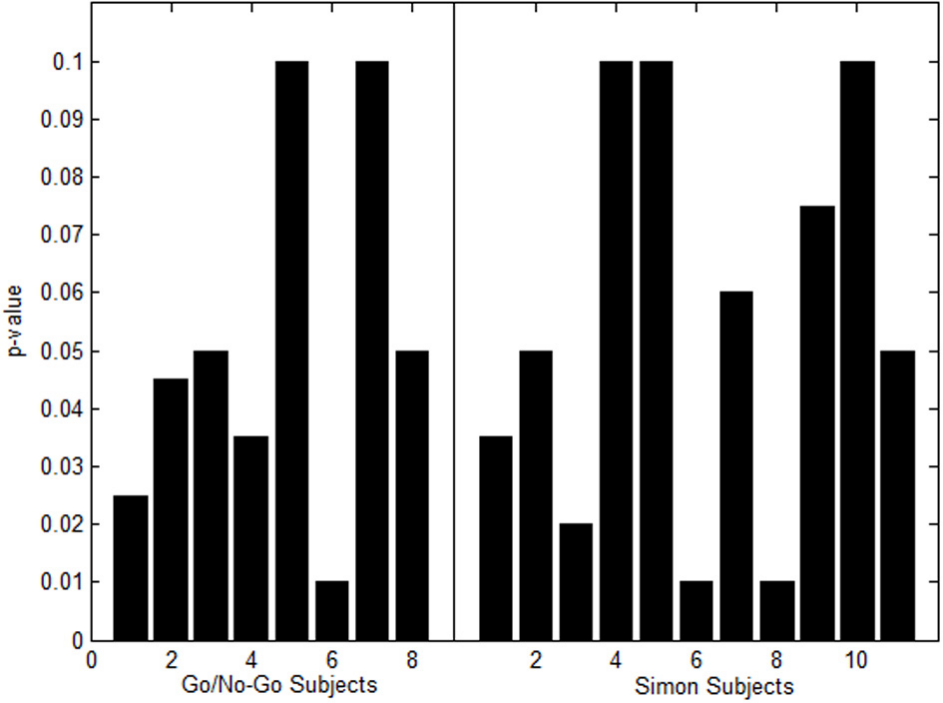
**Histogram of highest *p*-values to produce missing nodes in Go/No-Go Task (left) and Simon task (right)**.

For the Go/No-Go Task Dataset a *p*-value of 0.001 was set in the beginning. At this conservative value, 8 out of the 21 total subjects had one or more missing nodes. The *p*-value was increased in increments of 0.005 up to 0.1 Family-wise corrected. Figure 7 shows a histogram of the highest *p*-values needed for activation to appear in the subjects that had missing nodes.

**Figure 8:**
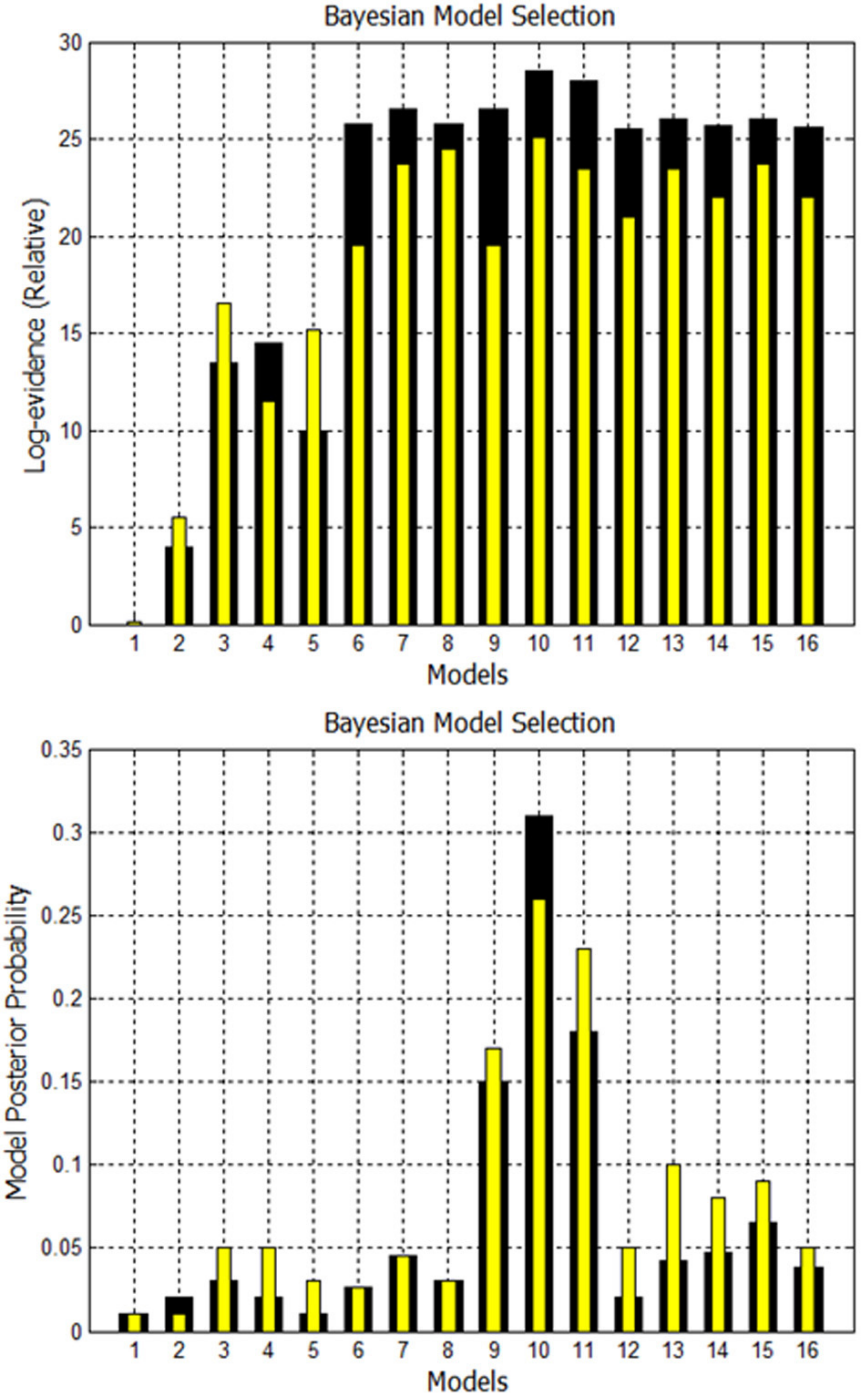
**Comparison of FFX analysis for Go/No-Go dataset for two techniques.** Black bars are for estimation of missing nodes with EM for this one subject. Yellow bars are for using the higher *p*-values to get the missing nodes. Model 10 has highest evidence in both cases, but other competing models are getting closer.

A comparison is made between using EM to estimate missing nodes and obtaining missing nodes by systematically increasing the *p*-value. Using both techniques, the evidence is computed for the 16 models shown in Supplementary Figure 1. The fixed effects analysis for Go/No-Go dataset for subject 1 is shown in Figure 8 where the relative log-evidence and maximum of the posterior (MAP) are plotted. The model posterior probability is highest for the 10th model when using either technique. However, both the relative log-evidence and the model posterior probability for the winning model (model 10) in model comparison are lower when using a higher *p*-value than when using EM-estimation. Also, the other competing models' posterior probabilities are increased when using a higher *p*-value compared to EM-estimation. This makes the distinction of the best model among other models more subtle and could eventually lead to selection of another model if the noise is high enough.

For Subject 10, the *p*-value had to be increased to 0.1 until the missing nodes were located. Subject 10 had the highest evidence for model 10 when using EM. However, when using a high *p*-value of 0.1, the highest evidence was no longer for the same 10th model. That was also the case for subject 18.

**Figure 9:**
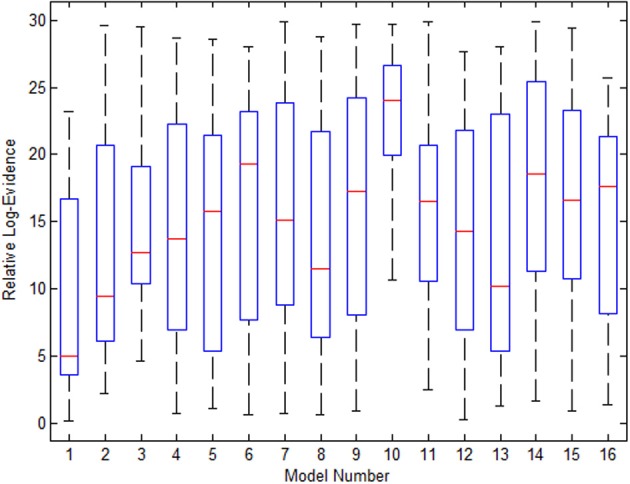
**The variance in the 16 models' evidence for the Go/No-Go dataset when using EM for estimation of missing nodes**.

Figure 9 shows the variance in evidence for the 16 selected models across the 21 subjects. The 10th model, which was most frequently the winner among the group, has the least variance and highest mean relative log-evidence.

For the Simon task dataset a *p*-value of 0.001 was set in the beginning. At this conservative value, 11 out of the 21 total subjects had one or more missing nodes. The *p*-value was increased in increments of 0.005 up to 0.1 Family-wise corrected. Figure 7 shows a histogram of the highest *p*-values needed for activation to appear in the subjects that had missing nodes.

**Figure 10:**
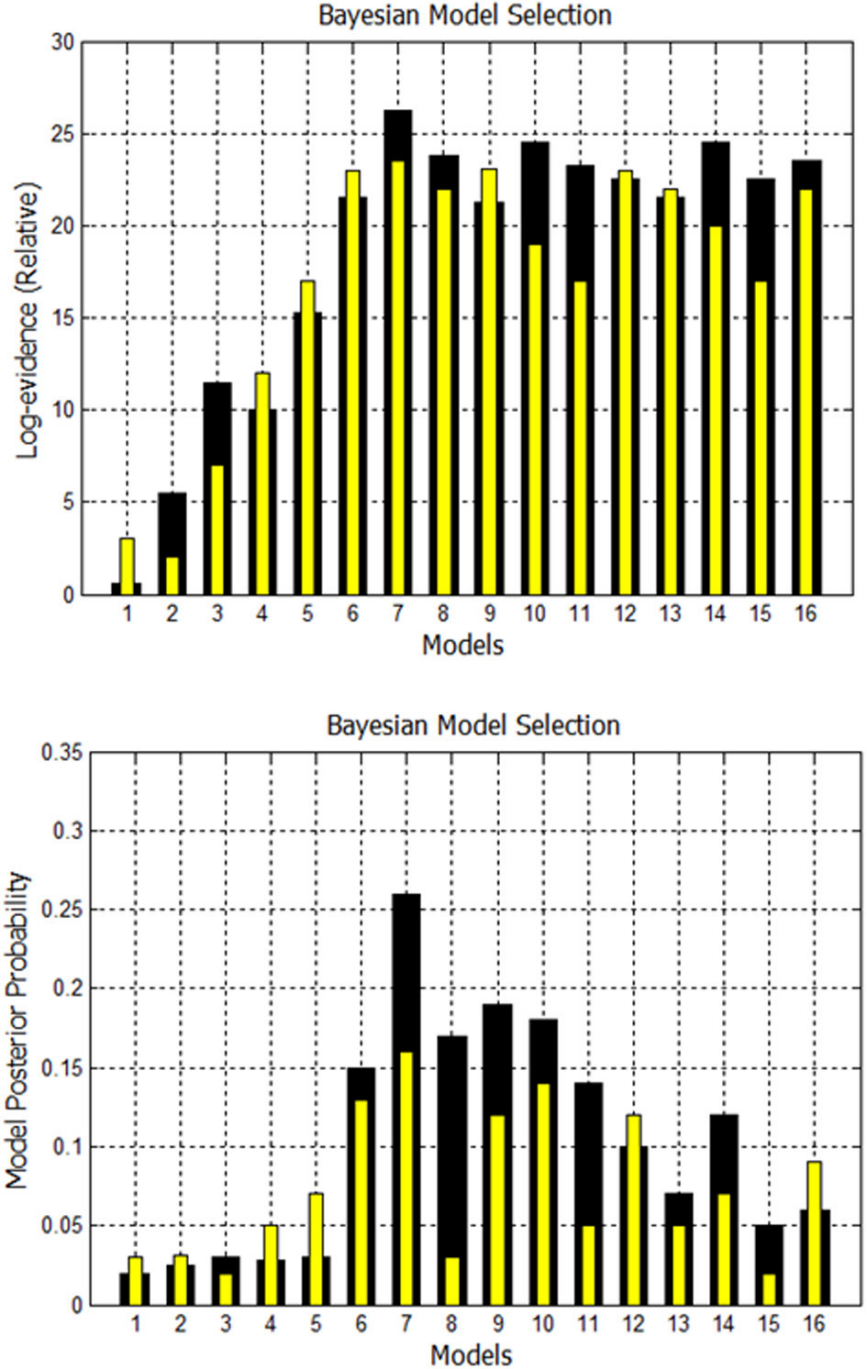
**Comparison of FFX Analysis for Simon task dataset for two techniques.** Black bars are for estimation of missing nodes with EM. Yellow bars are for using the higher *p*-values to get the missing nodes. Model 7 has the highest evidence in both cases for this subject.

A second comparison is made between using EM to estimate missing nodes and obtaining missing nodes by systematically increasing the *p*-value. Using both techniques, again, the evidence is computed for the 16 models shown in Supplementary Figure 2. The fixed effects analysis for Simon task dataset for Subject 3 is shown in Figure 10. The relative log-evidence and model posterior probability are highest for the 7th model when using either technique. However, both the relative log-evidence and the model posterior probability for the winning model (model 7) in model comparison are lower when using a higher *p*-value than when using EM-estimation. Also, the other competing models' posterior probabilities are increased when using a higher *p*-value compared to EM-estimation.

For Subject 8, the *p*-value had to be increased to 0.1 until the missing nodes were located. Subject 8 had the highest evidence for model 7 when using EM. However, when using a higher *p*-value of 0.1, the highest evidence was no longer for the same 7th model. That was also the case for subjects 10 and 17.

Although using a less conservative *p*-value appears to be a reasonable solution to missing node problem, it likely introduces non-task related information to the data. The previous experiments have shown that both the relative log-evidence and the model posterior probability for the winning model in model comparison are lower when using a higher *p*-value than when using EM-estimation. This direct comparison suggests that the introduction of unnecessary information can affect the computation of the evidence in a model comparison problem. Moreover, competing models posterior probabilities are actually shown to increase when using a higher *p*-value compared to EM-estimation which could eventually lead to selection of an incorrect model. For this reason we would recommend using EM estimation as opposed to a relaxed *p*-value to replace missing nodes in fMRI-DCM.

## Conclusion

This paper presents a solution to the problem of missing nodes in fMRI DCM subject groups contributing to the capacity of individual analyses. Based on sufficient evidence in the literature, the problem of missing nodes/regions in group studies is quite common (Penny et al., 2003a,b, 2010; Stephan et al., 2009; Rosaa et al., 2010). Based on personal communication, it appears that in exploratory analyses, most researchers would relax the *p*-value. However, when doing rigorous studies, they would resort to excluding subjects, which would not solve the personal phenotyping problem.

The main contribution of this paper was the introduction of missing data approaches as a prior step in DCM and dealing with the inconsistency in network structure, specifically the number of involved nodes. Missing data approaches have handled differences in node topology allowing computation of the model evidence of individuals in group studies. This approach is particularly useful when it comes to group studies, as the previously required manual filtering of the useless subjects and discarding of subjects who did not follow a given DCM model is no longer needed. A direct effect on clinical use, as presented, is the ability to classify individuals or patients. The limitation of not being able to test a certain subject in the context of classifying individual subjects' DCMs is no longer a setback. This increases the practical and clinical impact of a given study. Although DCM investigates the effective connectivity of a neuronal network, the limitation of studying directional influence on effective connectivity at the voxel-level still exists (Zuo et al., 2011).

Simulated experiments were designed and implemented to imitate a real dataset where the multiple topology problem existed. Solutions to the missing data problem included the simple zero-substitution and mean-substitution methods, and the more complex EM-estimation method. Given prior set ground truth, the highest classification rate was obtained when using the EM algorithm as opposed to the other methods. The complexity of EM was greater than using simpler methods (longer runtime although trivial), however a better classification accuracy of subject DCMs was achieved.

Feasibility was shown first using simulated subjects to model the variability in networks. Then the various missing data approaches were investigated. To assess the efficiency of the selected algorithms, classification tests were designed and carried out. These classification tests showed added ability in individual classification/labeling.

For practical purposes, the proposed usage of methods for data estimation in the context of DCM, real datasets were then used to verify effectiveness of the solution. Comparable results were indeed achieved and this was considered additional support to the validity of the solution to the problem. Similar patterns recurred for the two datasets justifying the efficiency of using missing data estimation methods as a means of forcing topological correspondence.

The usage of missing data approaches to estimate missing nodes in DCM, also showed a direct effect on the model ranking. The ability to include all subjects increased the model ranking capacity, when compared to not being able to include all subjects previously. Model comparison is an important benefit in both individual and group analyses. Model comparison was not possible when the model and subject had different topologies, particularly with regards to the number of active nodes in the two networks. It was shown that using missing data approaches on the model selection process and the eventual ranking of models to subjects had quite a positive effect. This was a vital contribution to individual analyses allowing the computation of the complete evidence matrix rather than having to assume no evidence or hindering the computation of evidence all together. Finally we recommend that EM-estimation be used rather than a relaxed statistical criterion to correct for missing nodes.

### Conflict of interest statement

The authors declare that the research was conducted in the absence of any commercial or financial relationships that could be construed as a potential conflict of interest.
