# Supplementary material for: Missing data estimation in fMRI dynamic causal modeling
Source: Front Neurosci. 2014 Jul 4;8:191. doi: 10.3389/fnins.2014.00191 (PMC4082189; doi:10.3389/fnins.2014.00191)
Supplement: Supplementary file 1 [file DataSheet1.DOCX]

***Supplementary Material***

**Missing Data Estimation in FMRI Dynamic Causal Modeling**

**Shaza B. Zaghlool^1^*, Christopher L. Wyatt*^2^**

^1^Biomedical Imaging Lab, Virginia Tech, Electrical and Computer Department, Blacksburg, VA, USA

^2^ Biomedical Imaging Lab, Virginia Tech, Electrical and Computer Department, Blacksburg, VA, USA

*** Correspondence:** Christopher L. Wyatt, Biomedical Imaging Lab, Virginia Tech, Electrical and Computer Engineering Department, Perry Street, Blacksburg, VA, 24060, USA.

[clwyatt@vt.edu](mailto:clwyatt@vt.edu)

Shaza B. Zaghlool, Biomedical Imaging Lab, Virginia Tech, Electrical and Computer Engineering Department, Perry Street, Blacksburg, VA, 24060, USA.

[shaza@vt.edu](mailto:shaza@vt.edu)

1. **Supplementary Data**

The EM algorithm is described as follows where the parameter vector $\theta^{i}$ is the current best estimate for the full distribution parameters and $\theta$ is the candidate for an improved estimate. The variable $i$ is the iteration counter and $T$ is the convergence criterion. Given the available data $X$, the missing data $Z$, and the unknown parameters $\theta$, along with the likelihood function $L\left( \theta;X,Z \right)=p(X,Z|\theta)$, the maximum likelihood estimate of the unknown parameters is determined by the marginal likelihood of the available data $L\left( \theta;X \right)=p\left( X | \theta\right)=\sum_{Z} p(X,Z|\theta)$. The EM algorithm finds the maximum likelihood estimate of the marginal likelihood by iteratively performing the following steps:

$$Begin initialize \theta^{0},T, i=0$$

$Do:i=i+1$

$E Step:compute Q(\theta;\theta^{i})$

$M Step:\theta^{i+1}=\arg\max Q(\theta;\theta^{i})$

$Until Q\left( \theta^{i+1};\theta^{i} \right)-Q\left( \theta^{i};\theta^{i-1} \right)\leq T$

$$Return \hat{\theta}=\theta^{i+1}$$

$$End$$

$Q\left( \theta;\theta^{i} \right)$is the likelihood of the data including the missing data $Z$ marginalized with respect to the current best distribution described by $\theta^{i}$. $Q\left( \theta;\theta^{i} \right)$ is the expected value of the log likelihood function with respect to the conditional distribution of the missing data given the available data under the current estimate of the parameters.

$$Q\left( \theta;\theta^{i} \right)=E_{Z|X,\theta^{(t)}}[\log L(\theta;X,Z)]$$

Given our noise-filling model, the likelihood function $Q\left( \theta;\theta^{i} \right)=p(X,Z|\theta)$ with $Q$ having a Gaussian form. The error being minimized is the sum of squares between $\theta^{i}$ and $\theta$. Once the algorithm converges, the final $\theta^{i}$ (which contains the distribution properties i.e. mean and standard deviation) is used to generate the missing data $Z$ so it can be used as input into the DCM.

**2. Supplementary Figures**

1

2

3

4

5

6

7

8

9

10

11

12

13

14

15

16

**Supplementary Figure 1.** Different models that were specified for Go/No-Go task. All DCMs are fully connected between all four regions (dotted lines). Inputs are indicated by the black boxes. The 16 models differ in their modulatory connectivity (solid lines).

1

2

3

4

5

6

7

8

9

10

11

12

13

14

15

16

**Supplementary Figure 2.** Different models used for the Simon task. All DCMs are fully connected between all three regions (dotted lines). The input to each model is indicated by the black boxes. The 16 models differ in their modulatory connectivity (solid lines).
